# Institutional Notes and News

**Published:** 1910-12-31

**Authors:** 


### Institutional Notes and News.

**Poplar Hospital for Accidents.**

The kind of fact which the public likes to know was brought out very well in the chairman's speech at the recent Festival Dinner of the Poplar Hospital for Accidents, at which Sir Owen Philipps presided. Since the Dreadnought was begun six months ago 345 accidents in connection with the building of it have been received by the hospital. This fact is likely to be appreciated fully by the Poplar Hospital authorities, since, as the Chairman also pointed out, the Hospital Committee is formed largely of the workmen who live in its neighbourhood. It is interesting and satisfactory, in other words, to see that the Poplar Hospital for Accidents is managed to such an extent by the classes who find it useful. Let us add that, whether for this reason or not, £3,759 was the total of subscriptions announced during the evening. Many distinguished people were present, among them: Lord Pirrie, Sir Charles Owens, Sir Edward Stern, Sir Montagu Turner, Vice-Admiral A. M. Field, F.R.S., Mr. C. Charleton, Mr. E. G. Saltmarsh, Mr. Robert Philipson, and Mr. C. F. Torrey, Sir Joseph Savory, Mr. J. C. Head, and Sir Edgar Speyer, President, the Hon. Sydney Holland, Chairman, and Mr. J. G. Broodbank, Acting Chairman of the Hospital.

**Cottage Hospital, Leatherhead.**

The Queen Victoria Memorial Cottage Hospital, Leatherhead, is in the happy position of congratulating itself upon the extensions which have been carried out during the past year. No fewer than ninety-two patients have been admitted, and there is a balance in hand of £163. The alterations were planned by Mr. F. R. Lee, of Great Bookham, and comprise as their principal part a women's ward extended to receive four beds. The operating theatre was much improved by the marble floor, which was a present from Mr. C. F. Howell. The above changes necessitated the closing of the hospital during August and September, but October saw it open again, with the exception of the large new ward, which was not then ready.

**City Hospital, Aberdeen.**

The old year was not allowed to come to an end at Aberdeen without the authorities of the City Hospital there deciding that tuberculous patients should receive the latest treatment and the proper accommodation which science and public opinion now demand. They have, therefore, decided shortly to open one of the reconstructed pavilions for consumptives. The chief factor, perhaps, in this decision has been the report of Dr. Matthew Hay, which was considered at a recent meeting of the Public Health Committee of the Aberdeen Town Council. He recommends the reconstruction of a pavilion to suit consumptive cases, firstly on statistical grounds, and secondly on educational grounds. The latter are the inculcation of the importance of ventilation, and the more precise information which breathing exercises, for instance, convey by their practice. It is suggested that when there is no demand for the pavilion for the use of consumptives, at other times it may be used for ordinary zymotic cases. Dr. Hay also lends his agreement with the proposal of the Local Government Board of Scotland for the notification of consumption, though he thinks notification, anyway at first, should be voluntary. Dr. Lister, of Newhills Sanatorium, has offered to serve as hon. physician when the proposed pavilion is ready for use.

**Worcester General Infirmary.**

At the meeting of the Executive Committee on December 19 it was decided to employ a local firm of auditors and to advertise in the local papers for applications. The committee then proceeded to discuss some adverse criticisms made at a meeting of the Co-operative Society on the institution. Three cases had been mentioned in which attention had not been promptly given. The main complaints were concerning inquiries which were made from time to time as to the suitability of certain applicants to be treated at the infirmary. A letter was read from the honorary medical staff recommending a statement to the effect that the committee reserved the right to inquire into the circumstances of any or every patient seeking treatment at the infirmary; the mere fact of being a subscriber to the institution did not entitle the person to treatment. It was pointed out that the infirmary was a purely charitable institution, and only for those whose circumstances did not permit of their obtaining private treatment. It was stated that most hospitals appointed an almoner, whose function it was to inquire into the circumstances of each patient, but at present the Worcester General Infirmary possessed no such officer.

